# SEM-EDS and hyperspectral images of vine leaves treated with antifungal products

**DOI:** 10.1016/j.dib.2025.111899

**Published:** 2025-07-18

**Authors:** Ramón Sánchez, Carlos Rad, Carlos Cambra, M.P. Castroviejo, Rocío Barros, Álvaro Herrero

**Affiliations:** a Grupo de Inteligencia Computacional Aplicada (GICAP), Departamento de Digitalización, Escuela Politécnica Superior. Universidad de Burgos. *Av*. Cantabria s/n. 09006 Burgos Spain; b Grupo de Investigación en Compostaje (UBUCOMP). Universidad de Burgos. Pl. Misael Bañuelos s/n. 09001 Burgos Spain; c Grupo de Investigación ICCRAM-EST. International Research Center in Critical Raw Materials for Advanced Industrial Technologies (ICCRAM). Universidad de Burgos. Pl. Misael Bañuelos s/n. 09001 Burgos Spain; d Servicios Centrales de Apoyo a la Investigación, Parque Científico Tecnológico (PCT). Universidad de Burgos. Pl. Misael Bañuelos s/n. 09001 Burgos Spain

**Keywords:** Precision agriculture, Hyperspectral imaging, SEM, EDS, Mildew, Copper, Sulphur, Vitis vinifera

## Abstract

Scanning electron microscope, better known by its acronym as SEM, is a very useful technique for obtaining high-resolution images of the surface of a sample. Hyperspectral imaging provides precise information for analysing vineyard vegetation that could help in improving pesticide application in precision viticulture technics. The present dataset is based on images of vineyard leaves, taken with both technics.

The leaves of the cv. Tempranillo, proceeding from a vineyard located inside of the Cigales Denomination of Origin, in north-central Spain, were treated with two Cu-containing products: ZZ Cuprocol (70 % w/v copper oxychloride) and Cuprantol Duo (14 % w/w copper oxychloride, 14 % w/w copper hydroxide). In addition, a contact pesticide widely used in intensive and traditional viticulture based on Folpet, copper-free but containing sulphur and chlorine, has been tested in its commercial form, Vitipec Blue (Cymoxanil 6 % w/w, Folpet 37.5 % w/w, Ascenza, PT).

Three dilutions were prepared, one of each compound, at the actual field application concentration of 1.33 g/L. The leaves were sampled and processed during the 2023 season. These leaves were taken from the central part of representative shoots of the vine canopy, with east and west exposures.

After the application of the pesticide dilutions, images of the leaves were taken with a 300-channel hyperspectral camera (Pika L, Resonon) using a mechanical bench synchronized with the camera. Then the SEM analysis was carried after prepare the samples.

Hence, such imagery is provided in the present dataset, based on the images taken from the leaves with both technics.

Specifications Table

**Table:** 

Subject	Earth & Environmental Sciences
Specific subject area	*Vineyard pest management. Hyperspectral imaging. SEM-EDS analysis.*
Type of data	Image, raw data, graphs
Data collection	Leaves subject of this dataset were collected during the 2023 season in a vineyard of the D.O. Cigales, in north-central Spain at 770 m above sea level. The hyperspectral camera used in the study is the Pika L model from Resonon (Resonon, USA), that covers the visible and near-infrared with 300 channels in a spectral range of 400 – 1000 nm. The camera has a spectral resolution of 2.7 nm and 900 spatial pixels, with a framerate of 64.96. The distance between the lens of the camera and the tray where the sample was located was 62 cm. The objective lens is a C-mount with a CMOS sensor type. A JEOL JSM-6460LV scanning electron microscope, with backscattered electron, secondary electron, and energy-dispersive X-ray detectors was used for image acquisition. The X-MaxN energy-dispersive detector is an area detector that allows elemental chemical composition analysis of a wide variety of samples using spot, area, mapping, and other methods of surface analysis. ED spectra were acquired using a single Oxford Instruments Ultim Max 20 SDD
Data source location	*(latitude 41° 49′ 17″ N, longitude 4° 35′ 49″ W),*
Data accessibility	***Please note:****All raw data referred to in this article must be made publicly available in a data repository prior to publication. Please indicate here where your data are hosted (the URL must be working at the time of submission and editors and reviewers must have anonymous access to the repository):* Repository name: Riubu Data identification number: http://hdl.handle.net/10259/10402 Direct URL to data: http://hdl.handle.net/10259/10402 Instructions for accessing these data: …
Related research article	

## 1 Value of the Data

**Fig. 1:**
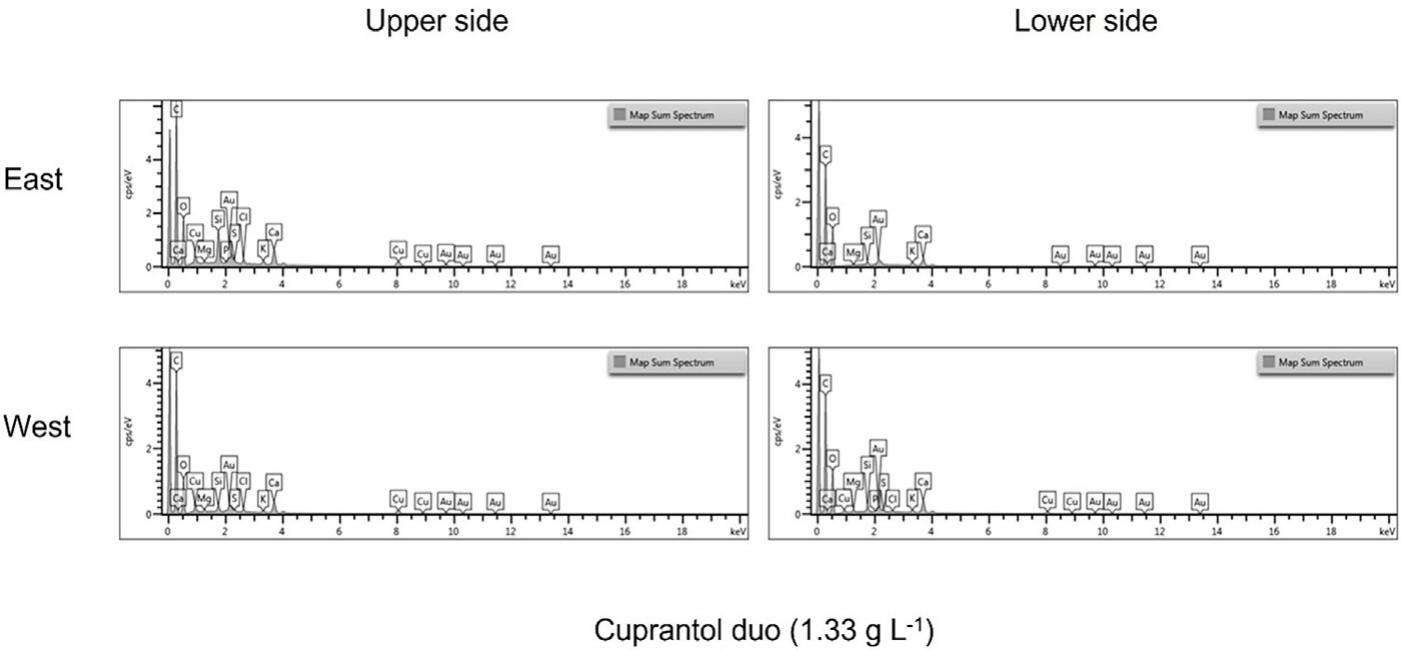
Different spectra of the elemental composition detected in Cuprantol droplets according to exposure and location on the vine leaf.

**Fig. 2:**
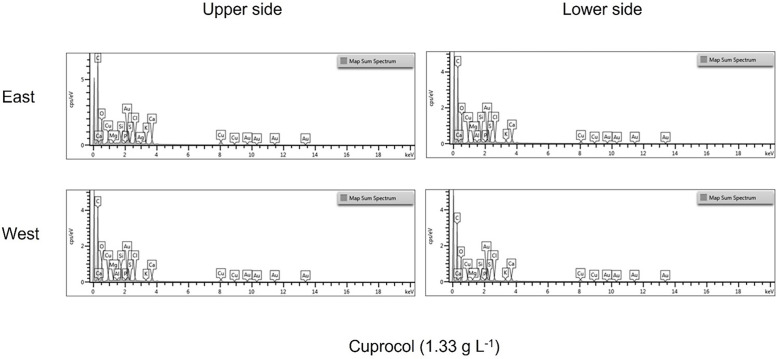
Different spectra of the elemental composition detected in Cuprocol droplets according to exposure and location on the vine leaf.

**Fig. 3:**
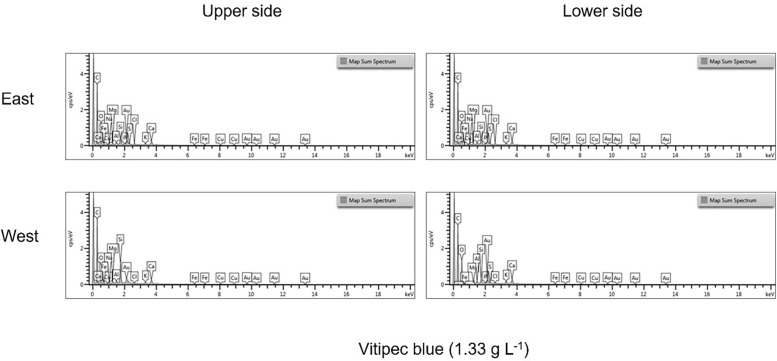
Different spectra of the elemental composition detected in Vitipec blue droplets according to exposure and location on the vine leaf.

Figs. 1, 2 and 3 show the spectra of the elemental composition that make up the product droplets. While the spectra for the copper-based products are similar across all four application methods. In the case of Folpet (Vitipec), application on the east side of the vine canopy provides greater element quantification than its quantification on the west side.

**Fig. 4:**
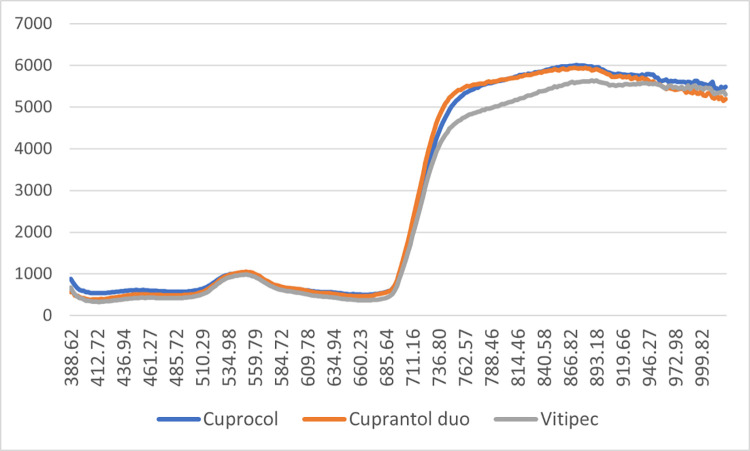
Average spectra of the product wet drops on the upper side of the leaf.

**Fig. 5:**
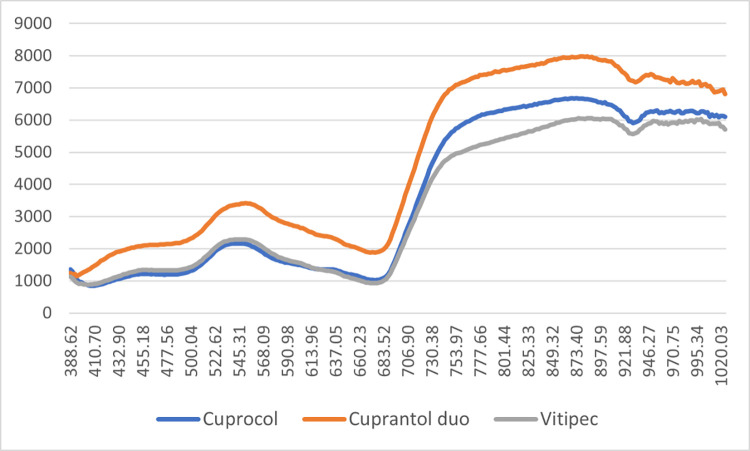
Average spectra of the product wet drops on the lower side of the leaf.

**Fig. 6:**
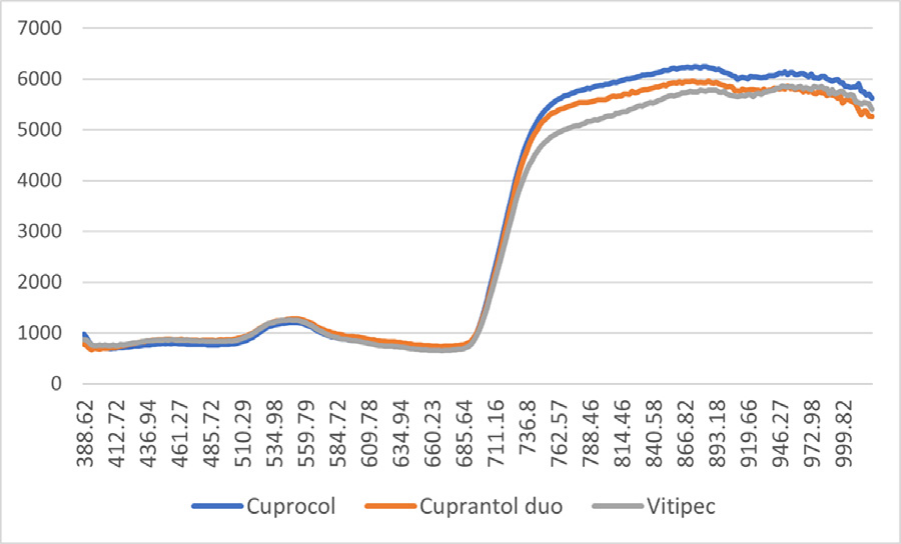
Average spectra of the product dry drops on the upper side of the leaf.

**Fig. 7:**
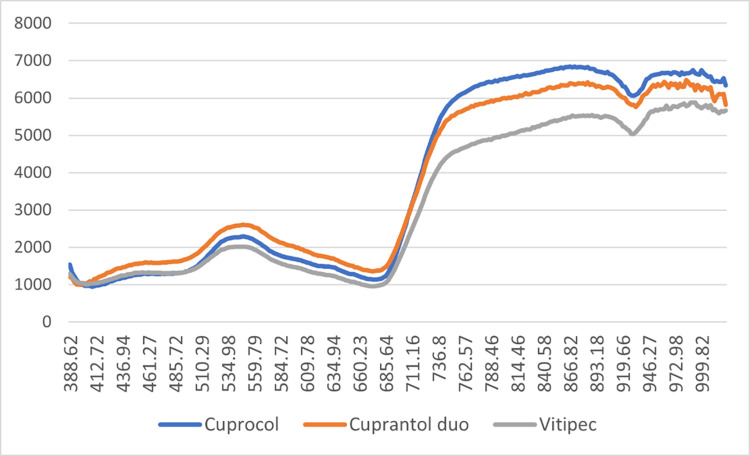
Average spectra of the product dry drops on the lower side of the leaf.

In the next Fig. 4, Fig. 5, Fig. 6, Fig. 7, the average spectra of the two drops of the product droplets for the same side of the leaf obtained from the hyperspectral images in wet and dry conditions were shown.

The spectra of the products deposited in the upper sides (Fig. 4, Fig. 5) show greater homogeneity between the products. In both cases (wet and dry), the spectrum of the sulphur-based compound displays lower reading values in the near-infrared region.

In the case of the products deposited on the lower side of the leaf (Fig. 6, Fig. 7), we observe more pronounced differences between the three compounds. The observed trend for Vitipec continues showing lower readings in the near-infrared spectral region. The spectral characteristics of the three products, both wet and dry, display different behaviour for each product and a greater variation in readings between them than that observed in the case of the products deposited in the upper side.

How can these data be reused by other researchers?

Other Researchers could use this data and images for develop its own investigation and go deeper in the detection of products presents in fungicides based on hyperspectral technics.

SEM-EDS images provide precise information on the distribution of the elements present in the drop of product, and the data in the csv file complete the information with the specific weight percentage, showed in the graphics of the Figs. 1, Fig. 2, Fig. 3.

The combined technics, data and images (SEM- EDS and hyperspectral), showed in this work open a path to determine not only the presence or absence of the fungicide products on vine leaves but also its concentration.

## 2 Background

For the proposal of this dataset, authors decided to choose two copper-based products that are used as broad-spectrum protectant fungicides in agricultural systems to control a series of plant diseases [1] worldwide. Also, a sulphur-based product (Vitipec Blue) has been tested.

Copper has proven to be the most effective compound against fungal diseases affecting vines and is therefore the most widely used compound in vineyards around the world [2,3]. Inaccuracies in the application of copper-based products can lead to its accumulation in soils and to economic and qualitative losses [4]. Further than this, recent studies determined that copper accumulation in soils contaminate the food chain, becoming a potential health risk for human health [5]. Correct application of copper-based products in vineyards is crucial to prevent the drifting to the soil.

Tempranillo is the fifth most grown grapevine variety worldwide, with a presence in approximately 17 countries and is the red variety more cultivated in Spain, according to the International Organisation of Vine and Wine [6].

Unlike conventional optical microscopes, SEM uses a focused beam of electrons to achieve higher magnification and resolution, which provides more detailed information about the sample specimens [7] . Additionally, coupling energy-dispersive spectroscopy (EDS) provides the elemental composition of the sample in images that show the detected elements separately. Studying the distribution of the elements that make up the fungicide product within the treatment droplet can help to a better understanding in how applications work and accurately determine their effectiveness. Combining hyperspectral images, which contain the spectrum of each pixel associated with the product droplet, with SEM-EDS images, that determine the weight percentage of each of the elements that make up the product droplets, as well as their distribution within them, could lead to develop effective methods of applying pesticide products in vineyards.

The validation of the spectral readings with SEM-EDS images that show the distribution of the different elements inside the drops of product could lead to a determination of the exact concentration of product in real time, so the viticulturist could know if they are applying the desired dose of compounds. Improving on the real-time information that the growers receive could be implemented, i.e., high increase of the detected concentration of the product, could be a symptom that the product tank is emptying. Also, it could be useful in automatized applications of product, allowing corrections of the nozzle angle in real time, to avoid drifting of product out of the leaf area.

The drops deposed on the leaves corresponding to the images in this database consist of five microlitres of the products placed in both upper and lower sides of vine leaves belonging to both exposition sides of the canopy (east and west).

The images are organized in 3 folders: ‘LEAF CLIPPINGS’, containing 4 RGB images of the treated areas of the leaves, cut with the 1 cm^2^ square die; ‘HYPERSPECTRAL IMAGES’, containing 8 images corresponding to the treated 4 leaves, with wet and dry treatment; and ‘SEM-EDS_IMAGES’, containing electron images, the images of leaf clippings corresponding to the detected elements, and the EDS image as result from the SEM- EDS analysis.

An CSV file containing the information of the specific composition of each drop has been included in the dataset.

The data are released on standard and open formats, so they could be used by researchers in order to perform further analysis of the different detected elements on the pesticide’s applications.

## 3 Data Description

The dataset includes hyperspectral and SEM-EDS images of vine leaves (*Vitis vinifera* L. cv. Tempranillo) taken from a vineyard located in D.O. Cigales (Valladolid, Spain) and treated with copper- and sulphur-containing pesticides.

The folder named “DATASET_HYPERSPECTRAL+SEM-EDS_IMAGES” contains three folders that make up the dataset.

**Fig. 8:**
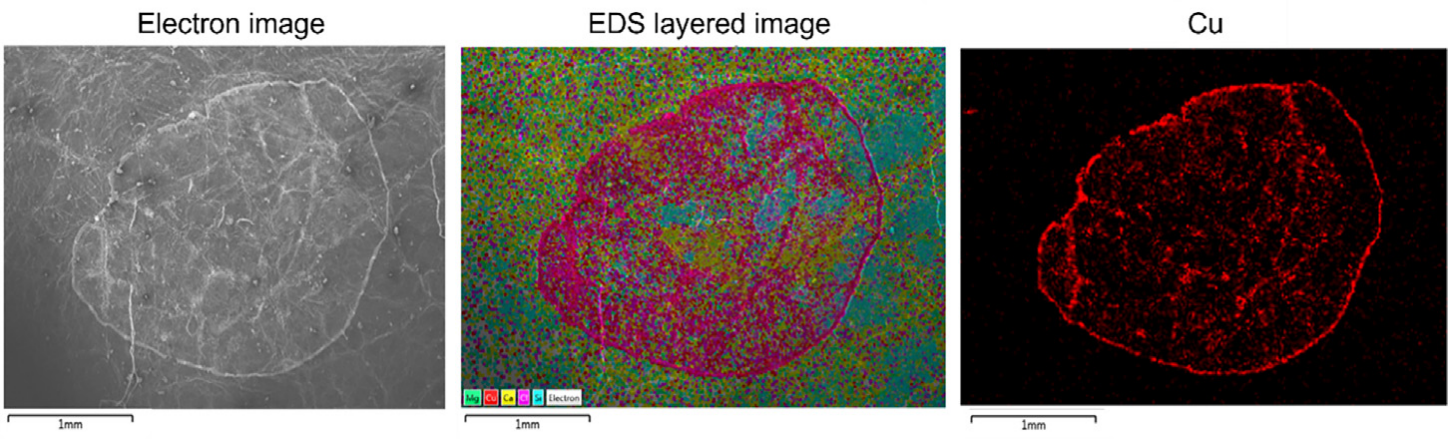
SEM-EDS images of the drop of Cuprocol, upper side of the leaf, west exposure.

The folder “SEM-EDS_IMAGES” contains twelve folders with the images of the product drops subjected to SEM-EDS analysis. Each of these folders is identified by the leaf exposure in the vineyard (E: east, W: west), the product (CUPROCOL: Cuprocol, CUPRANTOL: Cuprantol duo, VITIPEC: Vitipec Blue), and the side of the leaf where the product was applied (UP: upper side, LOW: lower side). Each folder contains images of the detected elements and a magnified image of the drop, called “Electron image”. Additionally, an image called "EDS Layered Image" corresponds to the image resulting from the sum of all the layers of the elements detected by the SEM-EDS. Examples of these images are shown in Fig. 8. A total of 185 SEM-EDS images are uploaded to this dataset.

The folder named “HYPERSPECTRAL_IMAGES” contains eight hyperspectral images corresponding to hypercubes in .bil format. Also, a .bil.hdr text file refers to each image, including all the metadata.

The folder named “CLIPPINGS” contains two folders named “EAST” and “WEST”, referring to the exposure of the leaves in the vineyard. Four images show the distribution of the leaf cuttings on the SEM cylinder.

The dataset is organized according to the structure shown in Table 1.

**Table 1:** Dataset structure.

HYPERSPECTRAL+SEM-EDS_IMAGES (3 folders)		
CLIPPINGS (2 folders)	EAST (2 images)	
	WEST (2 images)	
HYPERSPECTRAL_IMAGES (2 folders)	WET (4 images)	
	DRY (4 images)	
SEM-EDS_IMAGES (2 folders)	UPPER_SIDE (6 folders)	EUPCUPCOL (15 images)
		EUPCUPTOL (14 images)
		EUPVITIPEC (18 images)
		WUPCUPCOL (15 images)
		WUPCUPTOL (14 images)
		WUPVITIPEC (33 images)
	LOWER_SIDE (6 folders)	ELOWCUPCOL (15 images)
		ELOWCUPTOL (14 images)
		ELOWVITIPEC (16 images)
		WLOWCUPCOL (14 images)
		WLOWCUPTOL (14 images)
		WLOWVITIPEC (17 images)

## 4 Experimental Design, Materials and Methods

**Samples and analyses.** To generate the images appearing in this dataset, leaf samples were picked from a vineyard located in the Cigales Denomination of Origin, in north-central Spain (latitude 41° 49′ 17″ N, longitude 4° 35′ 49″ W. Leaves were taken in July 2023 from the central part of representative shoots of the vine canopy, picked from both sides of the canopy (east and west), and carried out to the laboratory under refrigerated conditions, at a temperature of approximately 10 °C. The leaves were treated with the three pesticides in the same morning they were collected to avoid dehydration. The sampled leaves were recollected at evening, around 21:00 pm., when the product is expected to be applied by viticulturist, avoiding de hours of more insolation.

**Product application.** The systemic Cu-containing antifungal products used for the study were ZZ Cuprocol (70 % w/v Copper oxychloride) and Cuprantol Duo (Copper oxychloride 14 % w/w, copper hydroxide 14 % w/w) both from Syngenta (Syngenta, CH). A contact antifungal compound, such is Folpet (2-[(Tricloromethyl)sulfanyl]−1H-isoindole-1,3(2H)‑dione), widely used in intensive and traditional viticulture and containing sulphur and chloride as heteroatoms, was also tested in the commercial form of Vitipec Azul (Cimoxanil 6 % w/w, Folpet 37.5 % w/w, Ascenza, PT). Detailed information of the products is shown in Table 2.

**Table 2:** Product specifications.

Product	Composition	Active element	Expected spectral range
ZZ Cuprocol	Copper oxychloride (36,458 % w/w)	Copper	723.98 nm – 758.27 nm [8]
Cuprantol duo	Copper oxychloride (14 % w/w) Cupric hydroxide (14 % w/w)	Copper	723.98 nm – 758.27 nm [8]
Vitipec Blue	Cimoxanil (6 %, w/w) Folpet (37.5 % w/w)	Sulphur	732 nm – 792 nm [9]

Products application was carried out on the upper and lower sides of sampled unwashed leaves. This application consists of localized depositions of 5 µL droplets of a 1.33 g *L*^−1^ solution of the products in distilled water, which corresponds to a real application dose of 400 g ha^−1^ of active ingredient of the product. Depositions are made using a precision micropipette in an area delimited with a marker on the leaf and identified with a number.

## 5 Experimental Setting

### 5.1 Hyperspectral imaging

The setup for taking the hyperspectral images that make up the dataset, consists of a mobile platform synchronized with the hyperspectral camera and the lighting device. A computer, which includes an external hard drive for data storage and the software to control the camera and the mobile platform, is disposed next to the acquisition stand.

### 5.2 Sample movement platform hardware structure and control device

The platform consists of two rails along which it moves slowly and synchronized with the hyperspectral camera, by means of the precise action of a stepper motor. The control software, based on Arduino, connects to the platform via USB, using a cable. This software allows defining the movement speed, selecting the start and stop locations for image acquisition, and synchronizing these processes in an automated way.

### 5.3 Illumination setting

The lighting system includes 4 halogen spotlights (USHIO Halogen, MR16) to improve the visible range. The lamps are mounted on 2 illumination rails attached to the camera support structure, with a beam angle of 25 degrees (figure X).

### 5.4 Hyperspectral image acquisition

The hyperspectral camera used in the study is the Pika L model from Resonon (Resonon, USA), that covers the visible and near-infrared with 300 channels in a spectral range of 400 – 1000 nm. The camera has a spectral resolution of 2.7 nm and 900 spatial pixels, with a framerate of 64.96. The distance between the lens of the camera and the tray where the sample was located was 62 cm. The objective lens is a C-mount with a CMOS sensor type.

### 5.5 SEM-EDS process

**Fig. 9:**
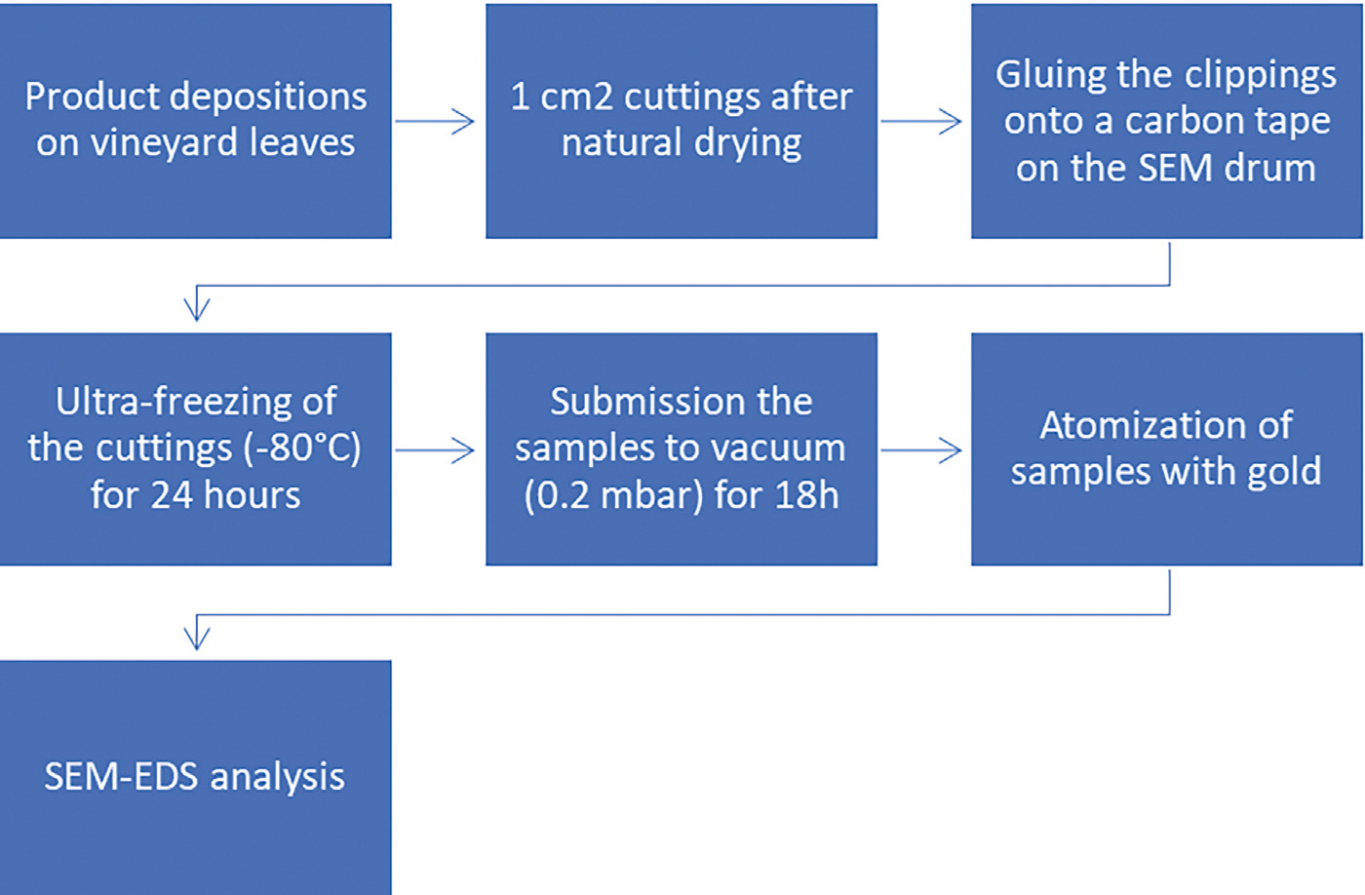
Sample preparation for SEM-EDS process.

As described in Fig. 9, the sample preparation for the SEM-EDS process starts with the product depositions with a precision micropipette. Then, after the natural drying of the droplets, 1 cm^2^ squares were cut around the depositions area using a die. The cut-outs are glued to the adapted circular base (drum) of the SEM, this preparation is taken to ultra-freezing (−80 °C) for 24 h. After the ultra-freezing process, the samples are submitted to vacuum of 0.2 mbar for 18 h. Gold atomization is necessary to increase the conductivity of the samples, before the analysis on the SEM microscope.

### 5.6 SEM-EDS acquisition

A JEOL JSM-6460LV scanning electron microscope, with backscattered electron, secondary electron, and energy-dispersive X-ray detectors was used for image acquisition. The X-Max^N^ energy-dispersive detector is an area detector that allows elemental chemical composition analysis of a wide variety of samples using spot, area, mapping, and other methods of surface analysis. ED spectra were acquired using a single Oxford Instruments Ultim Max 20 SDD

This equipment allows photographs at magnifications of up to x300,000. In our case, the vine leaf samples, since they are non-conductive, were metallized with Au using an Emitech K550X system with gold target. The specifications for the SEM-EDS image parameters are detailed in Table 3.

**Table 3:** Specifications of SEM-EDS images.

Resolution (Width):	512 pixels
Resolution (Height):	384 pixels
Image Width:	3.6mm
Image Height:	2.7mm
Stage Tilt Degrees:	0.23°
Specimen Tilt Degrees:	0.23°
Magnification:	35 x
Primary Detector:	51-XMX1002

## Limitations

The dataset is limited to specific products. More specific analysis should be carried on other vineyard cultivars and also evaluating the using of other products. Hyperspectral imaging has resolution limits in the images as the focus distance increase. We have also to point out that the preparations of the products in field situation could differ from the accurate concentrations prepared in the laboratory.

There is a potential variability depending on the specific surface of the leaf where the drop, is deployed. Also, some affections could vary the aspect of the leaf, such as diseases or different sources of metabolic stress, like the draught or nutritional causes. Climatic factors, like hail or sunstroke can also alter the leaf surface.

Complementary datasets could be generated by using other grapevine varieties and expanding the range of used pesticides.

## Ethics statement

The authors have read and follow the ethical requirements for publication in Data in Brief and confirm that the current work does not involve human subjects, animal experiments, or any data collected from social media platforms.

## CRediT author statement

**Álvaro Herrero:** Methodology, conceptualization, writing - reviewing & editing. **Carlos Cambra:** Methodology, conceptualization, software. **Carlos Rad:** Methodology, conceptualization, supervision. **Ramón Sánchez:** Data curation, formal analysis, investigation, writing - reviewing & editing. **Rocío Barros:** Conceptualization, writing - reviewing & editing. **M.P. Castroviejo:** Methodology, formal analysis, writing & reviewing & editing.

## Data Availability

RiubuSEM-EDS and Hyperspectral images of vine leafs treated with antifungal products (Original data).
